# Expression of auxin transporter genes in flax (Linum usitatissimum) fibers during gravity response

**DOI:** 10.18699/VJGB-24-05

**Published:** 2024-02

**Authors:** N.N. Ibragimova, N.E. Mokshina

**Affiliations:** Kazan Institute of Biochemistry and Biophysics of Kazan Scientific Center of the Russian Academy of Sciences, Kazan, Russia; Kazan Institute of Biochemistry and Biophysics of Kazan Scientific Center of the Russian Academy of Sciences, Kazan, Russia

**Keywords:** flax, Linum usitatissimum L., gravitropism, fiber, auxin transport, gene expression, лен, Linum usitatissimum L., гравитропизм, волокно, транспорт ауксина, экспрессия генов

## Abstract

Gravitropism is an adaptive reaction of plants associated with the ability of various plant organs to be located and to grow in a certain direction relative to the gravity vector, while usually the asymmetric distribution of the phytohormone auxin is a necessary condition for the gravitropical bending of plant organs. Earlier, we described significant morphological changes in phloem fibers with a thickened cell wall located on different sides of the stem in the area of the gravitropic curvature. The present study is the first work devoted to the identification of genes encoding auxin transporters in cells at different stages of development and during gravity response. In this study, the flax genes encoding the AUX1/LAX, PIN-FORMED, PIN-LIKES, and ABCB auxin transporters were identified. A comparative analysis of the expression of these genes in flax phloem fibers at different stages of development revealed increased expression of some of these genes at the stage of intrusive growth (LusLAX2 (A, B), LuxPIN1-D, LusPILS7 (C, D)), at the early stage of tertiary cell wall formation (LusAUX1 (A, D), LusABCB1 (A, B), LusABCB15-A, LusPIN1 (A, B), LusPIN4-A, and LusPIN5-A), and at the late stage of tertiary cell wall development (LusLAX3 (A, B)). It was shown that in the course of gravitropism, the expression of many genes, including those responsible for the influx of auxin in cells (LusAUX1-D), in the studied families increased. Differential expression of auxin transporter genes was revealed during gravity response in fibers located on different sides of the stem (upper (PUL) and lower (OPP)). The difference was observed due to the expression of genes, the products of which are responsible for auxin intracellular transport (LusPILS3, LusPILS7-A) and its efflux (LusABCB15-B, LusABCB19-B). It was noted that the increased expression of PIN genes and ABCB genes was more typical of fibers on the opposite side. The results obtained allow us to make an assumption about the presence of differential auxin content in the fibers of different sides of gravistimulated flax plants, which may be determined by an uneven outflow of auxin. This study gives an idea of auxin carriers in flax and lays the foundation for further studies of their functions in the development of phloem fiber and in gravity response.

## Abstract (ru)

Гравитропизм – адаптивная реакция растений, связанная со способностью органов растений располагаться и расти в определенном направлении относительно вектора силы тяжести. При этом асимметричное распределение фитогормона ауксина считается необходимым условием для тропического изгиба органов растения. Ранее нами были описаны яркие морфологические изменения флоэмных волокон с утолщенной клеточной стенкой, находящихся на разных сторонах зрелых участков стебля в области гравитропического изгиба. Настоящее исследование – первая работа, посвященная идентификации генов, кодирующих переносчики ауксина в этих клетках на разных стадиях развития и при гравиответе. В растениях льна идентифицированы гены основных переносчиков ауксина: AUX1/LAX, PIN-FORMED, PIN-LIKES и ABCB. Сравнительный анализ экспрессии этих генов во флоэмных волокнах льна, находящихся на разных стадиях развития, выявил повышенную экспрессию некоторых генов на стадии интрузивного роста (LusLAX2 (A, B), LuxPIN1-D, LusPILS7 (C, D)), на ранней стадии формирования третичной клеточной стенки (LusAUX1 (A, D), LusABCB1 (A, B), LusABCB15-A, LusPIN1 (A, B), LusPIN4-A, LusPIN5-A) и на поздней стадии развития третичной клеточной стенки (LusLAX3 (A, B)). Показано, что при гравитропизме повышалась экспрессия многих генов исследуемых семейств, в том числе отвечающих за приток ауксина в клетки (LusAUX1-D). Выявлена дифференциальная экспрессия генов переносчиков ауксина при гравиответе в волокнах, находящихся на разных сторонах стебля – верхней (PUL) и нижней (OPP): различие наблюдалось за счет экспрессии генов, продукты которых отвечают за внутриклеточный транспорт (LusPILS3, LusPILS7-A) и отток ауксина из клеток (LusABCB15-B, LusABCB19-B). Повышенная экспрессия PIN-генов и ABCB-генов была более типична для волокон OPP-стороны стебля. Полученные результаты позволяют сделать предположение о наличии дифференциального содержания ауксина в волокнах разных сторон стебля гравистимулированных растений льна, которое, возможно, определяется неравномерным оттоком ауксина. Исследование дает представление о переносчиках ауксина во льне и закладывает основу для дальнейшего изучения их функций в развитии флоэмного волокна и при гравиответе.

## Introduction

Plants are under the constant influence of abiotic and biotic factors, including unfavorable ones. The activity and coordinated action of auxin (indole-3-acetic acid, IAA) carriers in plants underlie a flexible network that mobilizes IAA in response to various environmental changes. This applies to plant tropisms as well. Regardless of the mechanisms involved, the activity of the phytohormone auxin is crucial for all tropisms, including gravitropism (Harrison, Pickard, 1989; Evans, 1991; Li et al., 1991; Rakusová et al., 2019). It has been shown that inhibitors of IAA transport block the development of the gravitropic reaction in plants (Li et al., 1991). The uneven distribution of auxin due to the participation of protein carriers during gravitropism is an important area of both fundamental and applied research related to plant lodging.

The implementation of the gravity response is associated with the formation of a gravitropic curvature. The formation of such curvature in plants occurs with the participation of different mechanisms: in young, actively growing organs (seedling roots, hypocotyles, coleoptiles), the bend is formed due to different rates of cell elongation on the different sides of the gravistimulated organ (Harrison, Pickard, 1989; Li et al., 1991; Zhu et al., 2019). On the other hand, the gravitropic curvature of mature stems, in addition to the above-mentioned mechanism (which, apparently, continues in the plant growing tip) in the parts of the stem that have ceased elongation, occurs due to the probable “contractile” properties of the fibers (Ibragimova et al., 2017). The mechanism of formation of gravitropic curvature of mature stems also has its own characteristics in plant species of different systematic groups: in angiosperm woody plants, it is formed due to changes in cambial activity and the formation of a tertiary cell wall (TCW) in xylem fibers on the upper side of the gravistimulated organ, leading to the formation of tension wood (Haygreen, Bowyer, 1996; Jourez et al., 2001). In mature gymnosperm stems, gravitropic curvature is provided by the formation of compression wood, which appears on the underside of the gravistimulated organ (Timell, 1969). Finally, in mature annual stems of herbaceous plants, including flax, gravitropic curvature is provided, as we assume, by primary phloem fibers having a cell wall (or TCW), while the formation of TCW is also observed in the xylem fibers of the upper stem side (Ibragimova et al., 2017). If, in the case of young organs, the role of auxin in the formation of curvature is actively studied, information on the distribution of IAA in mature organs is very limited and contradictory (Hellgren et al., 2004; Gerttula et al., 2015).

Auxin is distributed in the plant body by two different but interconnected transport systems: first, rapid flow in the phloem together with photosynthetic assimilates; and second, slow and directional polar transport of auxin from cell to cell (Adamowski, Friml, 2015). While phloem transport provides a general way of auxin delivery from its place of synthesis to the recipient organs, polar transport distributes auxin in an accurate manner, which is critical for the formation of local auxin maxima and is one of the key elements in its functioning (Friml et al., 2002; Zažímalová et al., 2010; Adamowski, Friml, 2015). Auxin carriers of the PIN family form the main part of this system, controlling the direction and speed of transport through a number of cells (Zažímalová et al., 2010). As for possible changes in the expression of genes for the PIN protein, the relevant data are currently limited to several model species.

In addition to the PIN (PIN-FORMED) family, auxin transport is carried out by other types of proteins: AUX1/LAX (AUXIN-INSENSITIVE1/LIKE AUX1), ABCB (subfamily of ATP-binding cassette transporters), PILS (PIN-LIKES), NRT1.1 (nitrate transporter 1.1), and WAT1 (WALLS ARE THIN1) (Manna et al., 2022). It is believed that at a low pH of the apoplast, auxin becomes protonated and can penetrate into the cell by diffusion. In certain types of cells, auxin can be transported to the cytosol by protein carriers, members of the AUX1/LAX family (Swarup, Péret, 2012). Inside the cell, auxin becomes negatively charged, and consequently, carriers are required to ensure its efflux, such as PIN and ABCB, through the cell membrane into the apoplast (Zažímalová et al., 2010). A less-characterized group of PILS transport proteins is probably responsible for intracellular auxin transport (Barbez et al., 2012).

In this study, the genes for the main auxin carrier proteins (PIN, AUX1/LAX, ABCB, and PILS) were identified in the flax genome. Their expression was evaluated using comparative transcriptomic analysis of the phloem fibers, which were sampled from control and gravistimulated flax plants. As a model system, we selected flax phloem fibers at different stages of development (with primary (PCW) and thickened tertiary cell walls (TCW)), as well as phloem fibers from different sides of gravistimulated flax plants at a late stage of TCW development. Flax phloem fibers are arranged along the stem axis in the bundles, which simplifies their isolation at different stages of development, separated in space and time (fibers reach a finite length during intrusive growth, and then layers of secondary and tertiary CW are sequentially deposited in cells (Gorshkova et al., 2003)). All this makes it possible to conduct diverse studies at the cell level. It was shown that during flax gravistimulation, phloem fibers localized on different sides of the stem (upper (PUL) and lower (OPP)) had morphological and structural biochemical changes (Ibragimova et al., 2017, 2020). Analysis of the expression of genes encoding the main auxin carriers in isolated fibers will reveal the type of auxin transport, which, as we assume, is activated in phloem fibers during gravity response.

## Materials and methods

Identification of auxin transporters. Using the Phytozome database, protein sequences containing functional domains (Pfam) PF01490, PF03547 and PF03547, PF00005, characteristic of AUX1/LAX, PIN/PILS, and ABCB auxin transporters, respectively, were identified. The identified genes for auxin carriers in flax were named in accordance with the orthologous sequence of Arabidopsis thaliana (thale cress); the functions of the products of all identified genes are predicted since their annotation is based on homology to the characterized genes of the thale cress. All sequences of the analyzed genes are presented in an Supplementary Material1.


Supplementary Materials are available in the online version of the paper: https://vavilovj-icg.ru/download/pict-2024-28/appx1.xlsx


Gene expression level and phylogenetic analysis. To evaluate gene expression, we used previously obtained transcriptomic data for flax plants (rapid growth phase), which are available in the FIBexDB database (https://ssl.cres-t.org/ fibex/flax/) (Mokshina et al., 2021). For analysis, phloem fibers were taken at different stages of development: before the formation of TCW (the stage of intrusive growth, iFIBa), at the early stage of TCW formation (tFIBa), at the late stage of TCW formation (tFIBb), as well as on different sides of the stem (PUL – upper part of bending plants, and OPP – opposite part) at the late stage of TCW formation during gravistimulation. Gravistimulation was carried out by tilting the plants (at the base) parallel to the soil (90 degrees). Gene expression in fibers at the late stage of TCW formation was analyzed 8, 24, and 96 hours after the plants were inclined. More than two-fold changes are being discussed.

To build a phylogenetic tree, the Maximum Likelihood method was used, the Le_Gascuel_2008 model (LG+G); Bootstrap support 1000. It was performed in the MEGA7 program.

## Results

The genes encoding the main families of auxin transporters were identified: AUX1/LAX, responsible for the influx of auxin into the cell (Swarup, Péret, 2012); PIN-FORMED and ABCB, responsible mainly for the auxin outflow (Zažímalová et al., 2010); PIN-LIKES (PILS), responsible for intracellular auxin transport (Barbez et al., 2012); expression of the listed genes was analyzed.

Identification and expression of LusPINs and LusPILS

In the flax genome, when searching in the Phytozome database (https://phytozome-next.jgi.doe.gov/) according to the presence of the PF03547 membrane transporter domain, 34 genes were found that correspond to 12 orthologs in Arabidopsis; a total of 15 genes with PF03547 (8 – PIN, 7 – PILS) were found in the Arabidopsis genome.

The sequence of the Lus10020829 gene (AT2G01420, PIN4) was corrected by us in the Augustus program (https:// bioinf.uni-greifswald.de/augustus/) and continued by the Lus10020830 sequence. The adjustment also took into account the results of BLASTX Arabi/Clami/Rice, available in the Phytozome database (JBrowse) (https://phytozome-next.jgi. doe.gov/jbrowse/index.html). Similarly, six more sequences were edited: Lus10009685 (AT1G73590, PIN1), Lus10002280 (AT1G71090, PILS2), Lus10018006 (AT1G73590, PIN1), Lus10036000 (AT1G71090, PILS2; Lus100360001 was excluded from analysis), Lus10042003 (AT1G73590, PIN1), and Lus10016704 (AT1G71090, PILS2). The Lus10004059 sequence (AT1G71090, PILS2) included a fragment of 119 unidentified amino acids (out of 443), which is probably due to problems with initial sequencing and/or genome assembly. Redundant domains were removed from the Lus10016688 sequence, after which the closest homologue to it was established as PILS7 (AT5G65980). Not typical domains were removed from the Lus10019229 sequence (AT1G20925, PILS1), but it cannot be considered fully predicted since it failed to establish the position of the start codon in silico. The Lus10012680 sequence (AT2G01420, PIN4) was increased from 231 to 517 amino acids. Sequences without predicted transmembrane domains were excluded from the analysis. When correcting the sequences, the integrity of some domains was restored, and the number of transmembrane domains and molecular weight approached the indicators characteristic of the members of the analyzed family.

Thus, after bioinformatics analysis and correction, 27 sequences remained out of 34, annotated as PIN membrane transporters, or PILS, which we used for further analysis (Table 1).

**Table 1.**
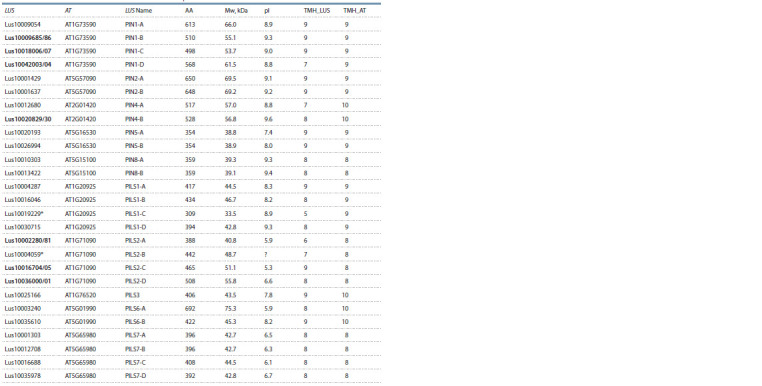
List and some characteristics of LusPIN and LusPILS sequences The corrected sequences are highlighted in bold. * Incomplete sequences. Hereinafter: LUS – Linum usitatissimum; AT – Arabidopsis thaliana; AA – number of amino acids; Mw – molecular weight, kDa; pI – isoelectric point; TMH – number of transmembrane domains.

The molecular weight of these proteins varied from 33.5 to 75 kDa, the value of pI – from 5.3 to 9.6; the number of transmembrane domains – from 5 to 10 (see Table 1).

**Fig. 1.**
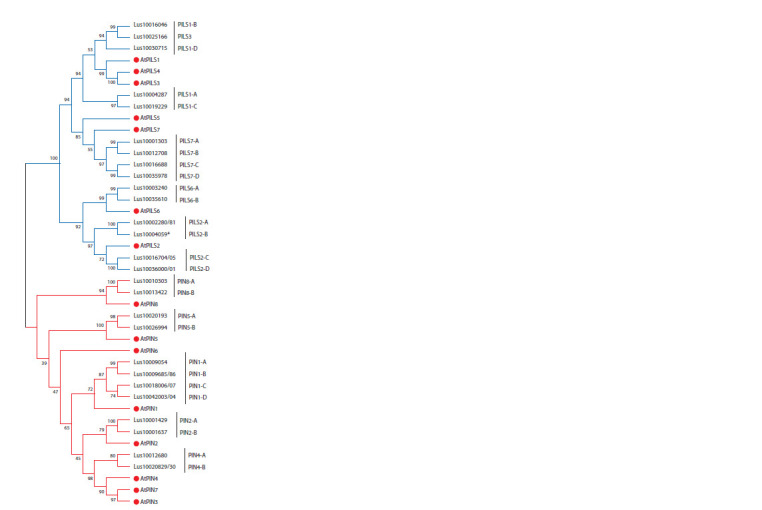
Phylogenetic tree for amino acid sequences PF03547 in A. thaliana and L. usitatissimum Maximum Likelihood method, model Le_Gascuel_2008 (LG+G). Bootstrap support 1000. Performed in the MEGA 7 program. The red marker indicates the sequences of A. thaliana.

To annotate the PIN/PILS genes, in addition to the BLAST results, we performed a phylogenetic analysis of the amino acid sequences of PIN/PILS in Arabidopsis and flax (Fig. 1). The analyzed sequences were expected to be divided into two clades: PIN and PILS. Several orthologs of the Arabidopsis PIN corresponded to two paralog genes in the flax genome (PIN2, 5, 8); four flax sequences were in the same group with the A. thaliana PIN1, and two L. usitatissimum sequences corresponded to the AtPIN3/4/7 group. Many PILS were also duplicated (see Fig. 1).

**Fig. 2.**
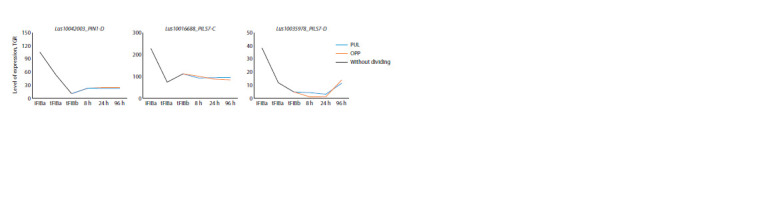
Expression of LusPIN/PILS with an increased level of expression only in intrusively growing fibers. Here and in Fig. 3–9: iFIBa – intrusively growing fibers; tFIBa – the early stage of tertiary cell wall (TCW) formation; tFIBb – the late stage of TCW formation. 8, 24, 96 hours – the time of fixation of the samples after the stem inclination; TGR – total gene reads; PUL – pulling side; OPP – opposite to the PUL-side. Without dividing – the plants were not subjected to gravistimulation, and the stem was not divided into PUL and OPP.

The expression of the genes Lus10001429 (LusPIN2-A), Lus10001637 (LusPIN2-B), Lus10004287 (LusPILS1-A), Lus10002280 (LusPILS2-A), and Lus10010303 (PIL8-A) was low (<16 TGR) and was not further analyzed. At different stages of development and during gravistimulation, 22 PIN/ PILS were expressed in the fibers. According to the dynamics of expression, several groups of genes can be distinguished. Interestingly, three genes (LusPIN1-D, LusPILS7-C, and D) showed an increased level of expression in fibers only at the stage of their elongation, while expression decreased in mature fibers, and there were no differences in fibers during gravistimulation (Fig. 2).

**Fig. 3.**
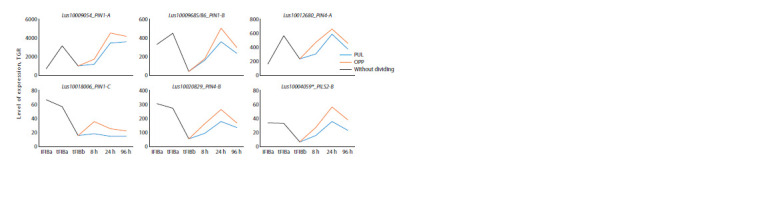
Expression of LusPIN1-A, B, C, LusPIN4-A, B, LusPILS2-B in flax fibers under normal conditions and in gravity response.

A group of genes was also revealed that had a pronounced expression peak in fibers at an early stage of TCW formation (tFIBa) (Fig. 3, LusPIN1-A, B, LusPIN4-A) or almost the same level in fibers during elongation and formation of thickened TCW (see Fig. 3, LusPIN1-C, LusPIN4-B, LusPILS2-B). At the same time, the expression of all these genes decreased in mature fibers and was activated again during gravistimulation, especially in OPP samples. The peak of expression during gravistimulation occurred at 24 hours, then expression decreased and was close to the minimum values characteristic of control tFIBb samples

**Fig. 4.**
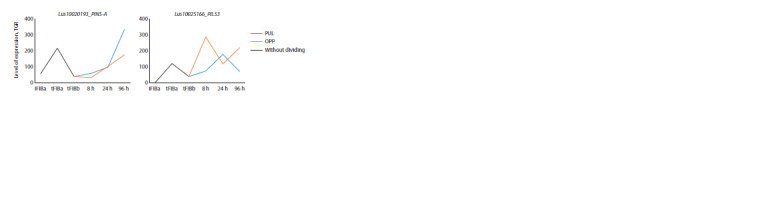
Expression of LusPIN5-A and LusPILS3 in flax fibers under normal conditions and in gravity response.

The expression of some genes did not change significantly in all samples (LusPILS2-C, and D) or was increased in fibers at an early stage of TCW formation (tFIBa) (LusPIN5-B, LusPILS1-C, D, LusPILS6-A, B, and LusPILS7-B), but did not differ significantly in fibers during gravistimulation (data not shown). Figure 4 shows the gene expression, the maximum value of which was observed during gravistimulation. LusPIN5-A had a maximum expression level in PUL fibers at 96 hours after gravistimulation. LusPILS3 had an increased expression level in OPP samples after 8 hours, while the peak of expression was also observed in PUL samples, but only 24 hours after the beginning of gravistimulation (see Fig. 4).

**Fig. 5.**
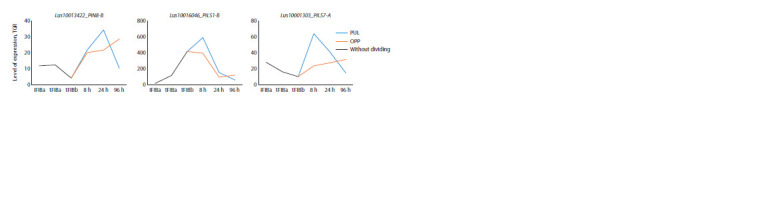
Expression of LusPIN8-B, LusPILS1-B, LusPILS7-A in flax fibers under normal conditions and in gravity response.

Three of the 22 genes showed an increased level of expression only during gravistimulation, and especially in PUL samples after 24 hours (LusPIN8-B), or 8 hours from the beginning of gravistimulation (LusPILS1-B, LusPILS7-A). The expression of LusPIN8-B decreased in PUL samples after 96 hours, but at the same time increased in OPP (Fig. 5). The most contrasting expression between PUL and OPP was demonstrated for LusPILS7-A, which was almost leveled after 96 hours.

Identification and expression of LusAUX1/LAX

Auxin influx carriers LusAUX1/LAX have a conservative PF01490 domain (Transmembrane amino acid transporter protein). In A. thaliana, the AUX1/LAX family is represented by four highly conserved genes called AUX1, LAX1, LAX2, and LAX3, which encode proteins similar to amino acid carriers (Young et al., 1999). In total, 82 genes of flax with PF01490 are represented in the Phytozome database. Of these, eight encode auxin transporters and correspond to three A. thaliana orthologous genes (AUX1, AUX2, and LAX3). All genes in this group encoded proteins close in molecular weight and isoelectric point; 10 transmembrane domains were predicted for all proteins (Table 2).

**Table 2.**
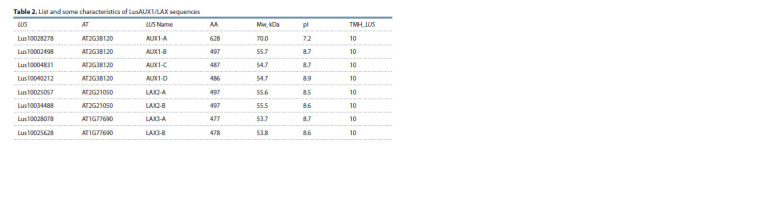
List and some characteristics of LusAUX1/LAX sequences

**Fig. 6.**
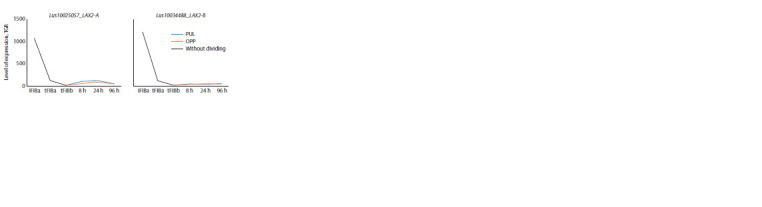
Expression of LusLAX2-A, B in fibers under normal conditions and in gravity response.

The genes encoding LusLAX2 (A and B) were highly expressed in intrusively growing fibers (iFIBa), while their expression dropped sharply in the fibers forming TCW and remained low during gravistimulation (Fig. 6).

**Fig. 7.**
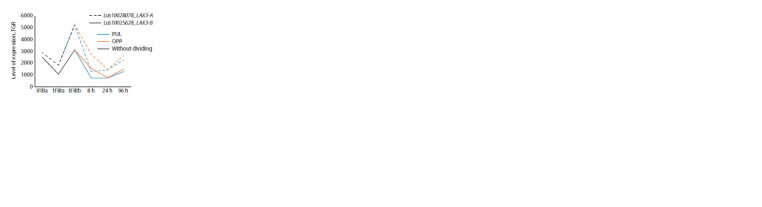
Expression of LusLAX3-A, and B in flax fibers under normal conditions and in gravity response.

The expression dynamics of LusLAX3 (A and B) were absolutely different from LusLAX2, while the expression dynamics between the two paralogs were identical, as in the case of LusLAX2. LusLAX3 had the maximum expression level in the fibers forming TCW at a late stage (tFIBb). During the gravity response, the expression level of these genes dropped sharply (Fig. 7)

**Fig. 8.**
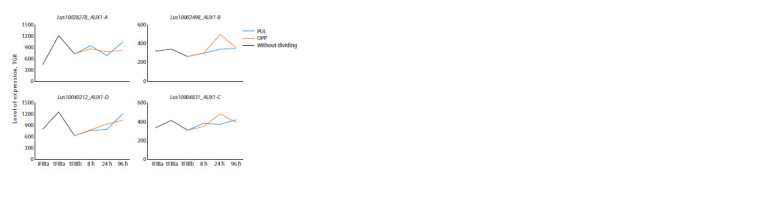
Expression of LusAUX1-A, B, C, D in flax fibers under normal conditions and in gravity response.

LusAUX1 genes had a relatively high level of expression in all samples, while four paralogs showed a clear division into two groups according to the expression patterns: with the maximum level in the fibers forming TCW (tFIBa, LusAUX1- A, and D), and with the maximum level of expression in the fibers of gravistimulated plants (OPP, 24 hours) (LusAUX1-B, and C) (Fig. 8).

Identification and expression of LusABCB

According to the Phytozome database, 206 ABC transporter genes are present in the flax genome, of which 32 genes belong to group B. To analyze the expression, we selected orthologs of the listed A. thaliana genes in flax. 25 genes corresponding to 5 Arabidopsis orthologs were identified: ABCB1 (2 flax genes), ABCB4 (4 flax genes), ABCB15 (9 flax genes), ABCB19 (8 flax genes), and ABCB20 (2 flax genes). The Lus10011977 sequence was partially corrected, and Lus10036616 and Lus10036617 were combined into one sequence (Table 3). Of the 24 genes, 4 (ABCB4 and 3 isoforms of ABCB15) had a low level of expression or were not expressed

**Table 3.**
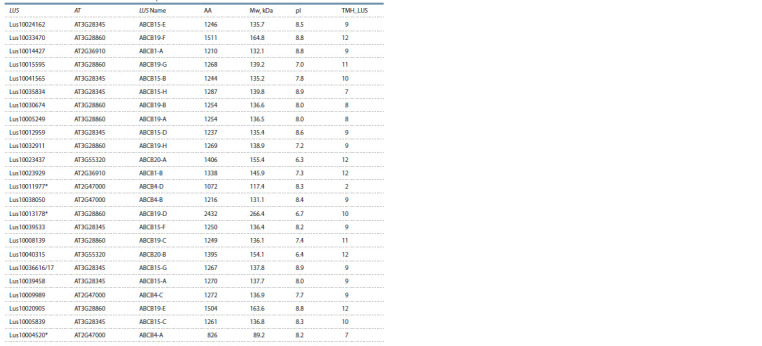
List and some characteristics of LusABCB sequences * The sequence may be incorrect.

The remaining 21 genes had different levels and patterns of expression. Thus, LusABCB20 (A and B) had a high level of expression in growing fibers, and at an early stage of TCW formation, in mature fibers, their expression decreased and almost did not change during gravistimulation. Two of the four isoforms of LusABCB4 had a maximum expression level in growing fibers, while the expression level itself was low, and the third isoform had a peak expression in fibers at an early stage of the formation of TCW. The most diverse expression patterns were characteristic of LusABCB19, which also had the largest number of expressed isoforms (8 genes) (data are not provided).

**Fig. 9.**
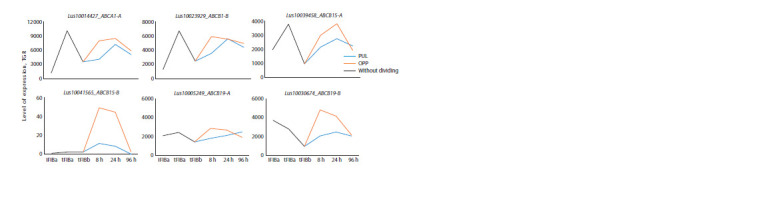
Expression of some LusABCB1, 15, and 19 gene isoforms in fibers under normal conditions and in gravity response.

We selected LusABCB as having the maximum difference in expression between PUL and OPP samples. Among the 6 genes, 4 genes had increased expression in the fibers forming TCW; the expression of these genes decreased in more mature fibers but increased in the fibers of gravistimulated plants, especially in OPP samples (significantly for ABCB15-B and ABCB19-B) (Fig. 9). A low level of expression was observed for LusABCB15-B, but the gene was specifically activated during gravistimulation and was practically not expressed in other samples. The expression of this gene is almost five times higher in OPP-side fibers compared to PUL (8, 24 hours) (see Fig. 9).

## Discussion

The action of auxin as a switch is closely related to the presence of local maximums and minimums formed in tissues (Adamowski, Friml, 2015). They are created, maintained, and modulated by intercellular auxin transfer, a plant-specific process. This process, called polar auxin transport, depends on the action of representatives of at least three auxin carrier families: PIN-FORMED, AUX1/LAX, and ABCB (Geisler et al., 2017).

In this study, the main auxin carrier genes were identified in flax plants: 12 LusPINs, 15 LusPILS, 8 LusAUX1/LAX, and 24 LusABCB. A comparative analysis of the expression of these genes in flax phloem fibers at different stages of development revealed increased expression of some of them at the stage of intrusive growth (LusLAX2 (A, B), LuxPIN1- D, LusPILS7 (C, D)), at the early stage of TCW formation (LusAUX1 (A, D), LusABCB1-A, B, LusABCB15-A, LusPIN1- A, B, LusPIN4-A, LusPIN5-A), and at the late stage of TCW formation (LusLAX3 (A, B)).

As known, all auxin transporters can be simplistically divided into three groups: responsible for the influx of auxin into the cell, outflow from the cell and intracellular transport. Two types of carriers participate in the outflow of auxin from the cell: PIN proteins and ABCB carriers (Zažímalová et al., 2010). PIN protein classification is usually based on phylogenetic relationships, subcellular localization, and the length of hydrophilic loop domains. From this point of view, members of the PIN protein family are usually grouped into three types: (1) canonical (PIN1, 2, 3, 4 and 7 – for A. thaliana), localized on the plasmalemma (PM), which mediate the intercellular flow of auxin; (2) non-canonical (PIN5 and 8 – for A. thaliana), which are localized on the EPR membrane and mediate auxin exchange between the cytosol and the EPR, contributing to intracellular auxin homeostasis; and (3) double, PM- and EPR-localized, PINs, such as PIN6 in A. thaliana, with unclear function (Zhang et al., 2020). According to the results obtained in this study, the following trend was observed for PIN carriers: the genes of canonical LusPINs were highly expressed in fibers at the early stage of TCW formation (see Fig. 3) and in fibers of gravistimulated plants, and their increased expression was observed in OPP samples (see Fig. 3), while increased expression of non-canonical PIN genes was observed in PUL samples (see Fig. 4, 5), which gives us the opportunity to assume a redistribution of auxin content in fibers on different stem sides during graviresponse. In confirmation of this assumption, a similar trend was also revealed in relation to ABCB carriers (see Fig. 9): a higher expression of LusABC1, 15, and 19 in OPP samples was shown. ABCB transporters carry out transport due to the direct binding of ATP and the energy that is released during ATP hydrolysis and can function when chemiosmotic gradients decrease or when auxin must move against the gradient (Zažímalová et al., 2010). Arabidopsis contains 21 full-sized ABCB genes (Kang et al., 2011), but only for four isoforms (ABCB1, ABCB4, ABCB19, and ABCB21) reliable data concerning auxin transport were obtained; for ABCB1 and ABCB19, data on coordinated action under gravitropism have been demonstrated (Geisler et al., 2017). It has also recently been shown that the pairs ABCB1/19 and ABCB6/20 represent the main ABCB auxin carriers over long distances through the vascular system in Arabidopsis (Jenness et al., 2022). There is an assumption that ABCB14 and ABCB15 are involved in auxin transport during stem lignification (Kaneda et al., 2011). It should be noted that the expression of genes for the ABCB transporter in Arabidopsis seedlings was studied in the roots, hypocotyl, and apex of the shoot (Geisler et al., 2017). It was assumed that ABCBs can play the role of the main auxin carriers: they are uniformly localized on PM, are usually found in various plant species, and persist stably regardless of internal and external signals. On the contrary, PINs are asymmetrically localized and dynamically distributed in response to endogenous and exogenous signals (Cho M., Cho H.T., 2013)

It should be noted that in this study, two paralogs homologous to the AtPIN3/4/7 clade were identified (see Fig. 1), which we annotated by the closest homologue as LusPIN4 (A and B). PIN3 is known to provide lateral auxin transport (Friml et al., 2002; Rakusová et al., 2019); high expression of PIN3 is shown in mature rami fibers with a thickened TCW (Bao et al., 2019), and during tension wood formation (Gerttula et al., 2015). In this study, the LusPIN4 genes (A and B) significantly increased expression during gravistimulation (24 hours) (see Fig. 3), but expression in the fibers of OPP samples was slightly higher compared to PUL samples. A similar trend was shown for gene expression and membrane localization of AtPIN3 and AtPIN4 during the hypocotyl apical hook formation, where pulling and opposite sides are also observed. The authors suggested that an increase in the content of PIN3 and PIN4 in the cell membrane on the opposite side is a decisive factor for the local auxin maximum formation (Zhu et al., 2019). It should be noted that we investigated the part of the stem of mature plants where the fibers do not grow by elongation, where the second (located below) curvature is formed. We have previously shown that when the upper part of the stem is removed (where the upper curvature is formed), plants implement the gravitropic reaction no less successfully (Ibragimova et al., 2017).

In the current work, it was shown that AUX1/LAX genes responsible for auxin influx into the cell increased expression during gravireaction to the level of expression in fibers at an early stage of TCW formation (LusAUX1-D for PUL), but there was no significant difference between OPP and PUL (see Fig. 8). However, a very interesting fact is that the highly expressed genes Lus10028078 and Lus10025628 (AT1G77690 – LAX3) had a single maximum in the control samples at the late stage of TCW formation (see Fig. 7). These data are consistent with the high expression of similar genes observed in mature rami fibers (Bao et al., 2019). With gravity response, the expression of these genes decreased and increased slightly towards the end of the reaction, approaching the values in the phase of the beginning of the formation of TCW (see Fig. 7). For LAX2, another effect was observed: the maximum expression in the fibers occurred in the phase of intrusive growth, but, as for LAX3 and AUX1, with gravity response, the expression values were again close to values at the early stage of TCW formation (significantly for the LusLAX2-A gene on the PUL side) (see Fig. 6).

The results of our study show that the increased expression of LusPILS in fibers quite often occurred during gravity response, both in the fibers of the PUL and OPP sides (see Fig. 4, 5), which suggests the presence of a relationship between gravity reaction and intracellular redistribution of auxin in general. At the same time, in some cases, a significant difference was observed in the fibers on different sides of the stem (LusPILS3 and LusPILS7-A) (see Fig. 4, 5). It should be noted that in LusPILS7-C and D, high expression was observed only at the stage of intrusive fiber growth (see Fig. 2). PILS transporters have been identified in silico as a putative family of auxin transport mediators; it has been shown that PILS, including PILS3 and PILS7, regulate the accumulation of auxin in the cell by retaining the added auxin (Barbez et al., 2012), which may also occur in the case of gravistimulation.

Thus, it was shown that during the gravitropic reaction, the genes encoding transporters responsible for auxin outflow from the cell (PIN and ABCB) and intracellular transport responsible for auxin homeostasis (non-canonical PIN5, PIN8 and PILS) were most significantly expressed. The expression level of these genes often approached the values that were characteristic of the stage at the beginning of TCW formation, when biosynthetic processes proceeded more intensively compared to those in mature fibers. It was shown that the expression of transporter genes responsible for the influx of auxin into cells (LusAUX1-D) also increased during gravitropism. The differential expression of the genes of the IAA carriers in the fibers located on different sides of the stem was revealed: the difference was observed due to the expression of genes, the products of which are responsible for intracellular transport (LusPILS3, LusPILS7-A) and auxin outflow (LusABCB15-B, LusABCB19-B). Increased expression of PIN genes and ABCB genes was more typical for the fibers of the OPP side of the stem.

## Conclusion

In this study, the main auxin carrier genes were identified in flax plants: 8 LusAUX1/LAX, 12 LusPIN-FORMED, 15 LusPIN-LIKES, and 24 LusABCB. The differential expression of the genes of the IAA transporters at different stages of development and in the fibers located on different stem sides during gravity response was revealed. We assume that the realization of this reaction may be associated with an asymmetric redistribution of auxin, mainly due to auxin intracellular transporters and transporters responsible for its outflow from the cell, an increase in the gene expression of which we observed during gravireaction. Further studies are required to clarify the mechanisms of auxin’s participation in the implementation of a gravity response not associated with growth by elongation, along with the participation of other hormones in it.

## Conflict of interest

The authors declare no conflict of interest.
